# The impact of food preservation on food waste

**DOI:** 10.1108/BFJ-02-2017-0114

**Published:** 2017-12-04

**Authors:** Wayne Martindale, Walter Schiebel

**Affiliations:** 1 National Centre for Food Manufacturing, Food Insights and Sustainability Service, University of Lincoln, Holbeach, UK; 2 Institute for Marketing and Innovation, University of Natural Resources and Life Sciences (BOKU), Vienna, Austria

**Keywords:** Consumers, Sustainability, Food waste, Frozen foods, Food preservation, Food value

## Abstract

**Purpose:**

The purpose of this paper is to demonstrate the relationship between food preservation and reducing consumer waste is of value in developing sustainable meal options. The research reports insights into Austrian marketplace for frozen and fresh foods that have been obtained from a consumer survey.

**Design/methodology/approach:**

The consumer survey methodologies indicate how preservation can change meal planning and lower food waste across frozen and fresh and ambient food purchases using freezing preservation methods.

**Findings:**

The results show food waste can be reduced by six-fold when frozen foods are compared with fresh foods.

**Research limitations/implications:**

This study highlights the requirement for a greater understanding of the probability that specific foods will be wasted with respect to the frequency of purchase. This is a limitation of the current study that has been investigated by other researchers.

**Practical implications:**

This research has enabled the identification of different food waste amounts for different food product categories. The data presented could be used to guide food product development so that less consumer waste is produced.

**Social implications:**

The research suggests a decision matrix approach can be used to can guide new product development and a model of this matrix is presented so that it may provide fit-for-purpose food preservation options for consumers.

**Originality/value:**

This paper will continue to highlight the overlooked value of food preservation during processing and manufacturing of foods and their preparation in households.

## Introduction

Consumers produce the greatest amount of food waste and loss in the food supply chains of developing and developed economies (Gustavsson *et al.*, 2011). A recent pan-European food waste programme has identified consumer food waste as a major challenge (COST Action TD1203, EUBIS). The COST Network, EU network on food waste valorisation has given attention to solving the amount of consumer food waste produced through technological and policy interventions (Morone *et al.*, 2017; Privett *et al.*, 2016). Reducing all food losses will result in a more secure global food system and it is important for us to show how consumers can reduce food waste in households. This is where food preservation has an important role in facilitating this waste reducing action because it improves the utilisation of food. It has also been identified that understanding why food is wasted by consumers during meal occasions develops of waste reduction strategies that can be used for different foods and preservation methods (Martindale, 2014).

Previous food waste reduction initiatives have typically focussed outside of this consumer arena and they have focussed on manufacturing and retail food losses. They have been successful at designing out food waste using the right-weighting of food products (portion control) and light-weighting of packaging (material resource efficiency). Their success has been made possible through cooperative actions across the food industry that have developed joint responsibility for food waste. It is essential that these initiatives now act to reduce the food that consumers purchase but do not eat (Mena *et al.*, 2011). Furthermore, FAO reported Food Balance statistics show supply chain losses for food groups such as meat, fruit and vegetables to be below 5 per cent of production or domestic supply quantities (Martindale, 2017). While these food losses remain incredibly important it is reported by national agencies and government departments that consumers’ food waste regularly reaches 20 per cent or more of food purchased (Defra, 2017).

There has been an emergence of re-distribution schemes and community focussed actions that have been successful at removing food waste from supply chains. Redistribution of foods that are close to shelf-life limits and schemes that facilitate providing food to consumers such as “community fridges” have an exceptionally important role to play in waste reduction particularly where communities experience limited accessibility and affordability of foods. The redistribution of foods from retailers and manufacturers that are close to shelf life limits or charitable donations has also seen the impact of using on-line communication technologies that connect providers with consumers of redistributed foods (Aschemann-Witzel *et al.*, 2017; Aschemann-Witzel *et al.*, 2015). What has become evident in this arena is the reduction of food wastes from the food supply chain to the point of consumer sale is dependent on the application of many actions. That is, there is no single solution here and many actions that redistribute, involve communities and use on-line technologies will help to reduce food waste and create awareness of responsible use of foods. The study reported here highlights the value of preservation technologies and the need for food category models that take account of differing shelf life and quality considerations because these will help to guide food policy. Previous studies of fresh and frozen shelf life of foods have shown a reduction in household waste associated with frozen food use (Martindale, 2014). A more recent study in the Netherlands has developed a stochastic model to show the influence of ambient, frozen and fresh preservation on household food waste (Janssen *et al.*, 2017). This study is critically important because it shows how food preservation methods that extend shelf life of foods in the home can reduce food waste over annual time periods. These studies also suggest that knowledge of food preparation and the best use of foods in households are critical in waste reduction.

Schemes that engage and redistribute resources to reduce food waste do not fully address the issue of food and drink products being wasted by consumers because they are not designed to reduce food waste. They redistribute food that would otherwise be waste; the study reported here focusses on reducing the wastage of food that is purchased with the intention of using it. The preservation of foods and types of food preservation methods available to consumers can facilitate this because it reduces food degradation and improves the utilisation of food in the domestic environment. This is a principle that has remained largely unconsidered even though the production of food waste increases greenhouse gas emissions or the carbon footprint of food consumption (Garnett, 2013; O’Rourke, 2014). It is crucial to consider food waste reduction as an outcome of using preserved foods because research carried out previously demonstrated it can help us to define the sustainability of meals that consumers prepare (Martindale, 2017).

In this study, it is demonstrated how frozen preservation can provide greater utilisation of food by consumers and reduce household food waste. It is not intended to show frozen is the only option for reducing consumer food waste. It is hoped that the research will highlight the use of preservation methods in reducing consumer food waste and that there are several factors that must work together in food waste reduction is to be successful. Previous research carried out in the UK market compared fresh and frozen food use in households and the amount of consumer food waste was dependent on food preservation method. The study showed a 47 per cent reduction in household food waste for frozen products compared to fresh products (Martindale, 2014).

Frozen food in this study is defined by all food that is frozen via quick freezing; this ensures the cell intactness and preserves the nutritional value of the food. The process of freezing food in this household focussed study is defined as non-frozen food which gets frozen via a standard freezer (at home), as such this is slow freezing where cell structure is not maintained and it is less beneficial than quick freezing but adds to shelf life significantly. The definition of fresh food in this study is all non-frozen and non-freezing food.

Working with frozen foods not only gives us an opportunity to consider the value of food preservation in households but we must also consider manufacturing factories providing efficient use of resources and continual availability (Tukker, 2015). This provides us with the opportunity to develop models of food preservation that identify control points in the supply chain that can maximise food waste reduction. Frozen and freezing foods define this requirement more effectively than many other food supply chains that do not preserve foods. The consideration of frozen or freezing foods in this study has provided an opportunity to investigate these wider impacts on food resource use by consumers. For example, freezing foods provides availability of out-of-season produce which can be included in the sustainability assessments of frozen and fresh produce (Foster *et al.*, 2014). While these benefits of food preservation are important it is the impact on consumer food waste that is investigated here. The value of localising food supply is important in the sustainability arena if it can provide what consumers demand and increased resilience. There are studies that show localising food supply can achieve this, particularly where there are strong regional food identities and a cultural preference of using food service (Caputo *et al.*, 2017). Localisation and the value of it to the food system are not within the scope of this current study even though it is important to consider food preservation has enabled the supply of foods that are out of season to consumers. Indeed, this was why preservation of fruits and vegetables using pickling and osmotic preserving emerged traditionally (Martindale, 2017).

Frozen foods have played a pivotal role in enabling the global food supply chain to evolve and without that food losses would be increased in agriculture and processing. Many of the food supply chain issues highlighted in current food loss and food waste research do not exist with frozen foods because quick freezing leads to the extended shelf life gains that many waste reduction initiatives seek (Parfitt *et al.*, 2010). Furthermore, freezing keeps within the conditions of “clean label” labelled trends and often provides greater portion control in the home (Shove and Southerton, 2000). The “clean label” trend is now clearly identified in retail environments where there are demands for ingredient labelling that clarifies ingredients and communicates any potential allergens introduced in processing and manufacturing (Asioli *et al.*, 2017).

The Austrian market research reported in this paper allows us to extend current understanding of the utilisation of frozen foods. It also leads us to consider the broader issue of what incentivises consumers to eat a more sustainable diet. Austrian households currently produce around 369,000 tons of packed and unpacked food waste each year and there is over 23.4 million tonnes of food waste produced by households across the EC member nations (Bräutigam *et al.*, 2014; Stenmarck *et al.*, 2016). A sustainable diet must eliminate this food waste, the Austrian food waste volume is equivalent to 300€ of food thrown away per household year (Lebersorger and Schneider, 2011; Penker and Wytrzens, 2005). The data presented here shows both frozen food purchases and household freezing decrease food waste significantly and this has important implications for providing sustainable meals and diets.

## Research method

The Austrian market data was collected via an online survey carried out by the Institute of Marketing & Innovation, University of Natural Resources and Life Sciences, Vienna (BOKU) and Gesellschaft für Konsumforschung (GfK SE) during July 2015 (GfK, 2016). The survey questionnaire obtained data from 2,800 participants on the frequency of their food purchases for fresh and frozen foods.

The survey participants were selected to represent the typical Austrian population with regard to age and educational level. The selection made for geographic distribution across the Federal States was proportional to the population in each Federal State. The selection to the panel of 2,800 was made using the GfK market survey methods used for market research. GfK are a commercial and international company that provided the survey panel of 2,800 households. GfK’s services are routinely used by the food sector by manufacturers and retailers to develop business activities and identify food and drink trends. The participants used in this survey bought food and drink for their household and were asked how much food they wasted across six food groups as a percentage of the total amount of the food they purchased. The six food groups were selected because they were important food categories in Austria that have both frozen and fresh options. Notably this included bread where the offer and purchasing of frozen bread rolls is typical for Austrian consumers.

The participants of the survey were asked to consider their household food waste in a week from the food they purchased, partly utilised food, leftovers (plate waste) and preparation residues. The core questions of the survey that asked participants to report their proportion of food purchased that was wasted as a percentage were as follows:

1. What percentage of fresh food from your household purchases do you throw away?
2. What percentage of the frozen food from your household purchases do you throw away?
3. What percentage of fresh food from your household purchases do you throw away per following product groups?
4. What percentage of frozen food from your household purchases do you throw away per following product groups?

The food groups were fruit; vegetables (including specific questions for potatoes and spinach); bread (fresh only); pasta; meat; and, fish (fish sticks also known as fish fingers for frozen foods). The core questions were developed in terms of what food product groups were wasted in households. The survey also collected demographic information so that the 2,800 participants reflected a typical sample of the Austrian population and this was determined using GfK’s demographic methods.

## Research results

**Figure 1:**
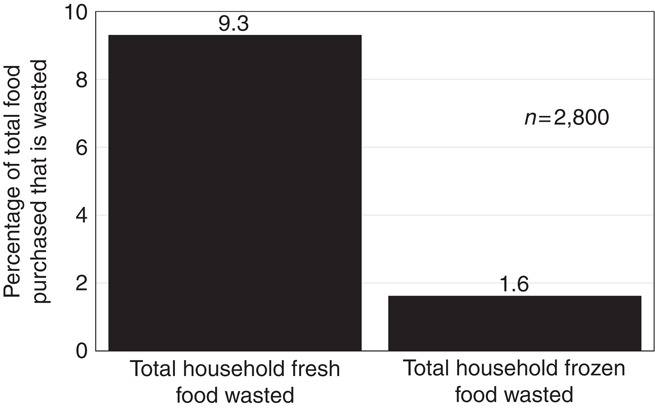
The amount of food waste associated with the total purchases of fresh and frozen foods in Austrian households

The amount of food waste produced in the sample of 2,800 Austrian households is shown in Figure 1. The data show that participants reported wasted 9.3 per cent of total fresh food purchased and 1.6 per cent of total frozen food purchased. Thus, the amount of reported food waste derived from the fresh foods is 5.8-fold greater than that of frozen foods in the 2,800 households assessed. This means that the six fresh food groups have a reported food waste that is 5.8-fold greater than comparable frozen food groups (see, Figure 1).

**Figure 2:**
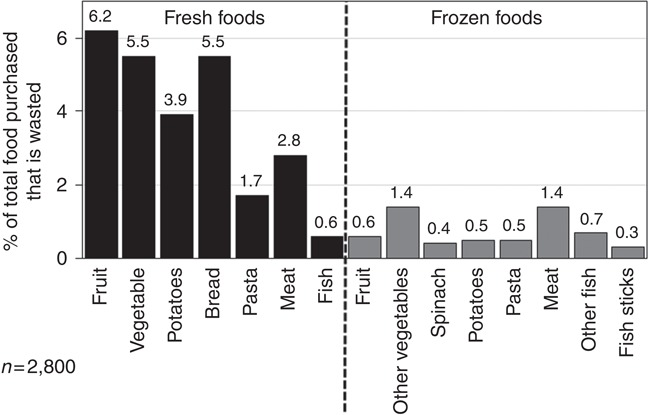
The percentage of food purchases wasted for the fresh and frozen food product categories assessed

Figure 2, shows the food waste for fresh and comparable frozen food groups assessed in the Austrian study of 2,800 households. The food groups are fruits, vegetables, bread, pasta, meat and fish. Data obtained for the vegetable group were also specifically obtained for potatoes and spinach because of the importance of these products in the frozen categories. A similar approach was taken for fish products where fish sticks (also known as fish fingers) are an important frozen product category.

Figure 2, shows the amount of food waste derived from fresh food purchases is greater than frozen food purchases across the six food groups assessed apart from fish which is assessed as “other fish” in the reported frozen products here. These data are summarised in Table I where the ratio of fresh to frozen food waste is provided.

## Research analysis

The goal of the research reported is to show how food waste behaviours connect many sustainability issues across the complex food choices consumers make when meals are prepared. Our research shows food manufacturers and food retailers occupy critical points in supply that can determine how these food consumption behaviours can be transformed into more sustainable ones. An important way of achieving this is through reducing the food waste associated with every meal.

Figure 1, shows fresh foods purchased have a reported 5.8-fold greater food waste compared to frozen food purchases in a survey of 2,800 Austrian households. The assessment of waste from different food groups provides important insights into how households utilise fresh and frozen foods (Figure 2). Table I, shows the ratio of fresh to frozen food waste across the food groups shown in Figure 2. It can be seen that fresh food is wasted in greater amounts than frozen food in every category except fish where fresh food waste is 0.9 of frozen food waste. The ratios show that the greatest differences between fresh and frozen food groups are seen for fruit where fresh is 10.3-fold greater than frozen and potatoes where fresh is 7.8-fold greater than frozen.

Notably, the fresh to frozen ratio of specific food products (Figure 2), include fresh vegetables and frozen spinach which is 13.8; and, for fresh fish and frozen fish sticks (also known as fish fingers) it is 2.0 in Austrian households. Spinach and fish sticks are specifically tested here because they are extremely popular for meal purchases in the Austrian and other European marketplaces. The 13.8-fold greater fresh vegetable waste than frozen spinach waste; and 2.0-fold greater fresh fish waste than fish stick waste is important because these products are developed to be directly placed into meals. They emphasise the impact of food product development when it is aligned to the portioning of food in meal preparation and if this is made to be optimal there is less food waste. This relationship between method of food preservation and portioning is also apparent with other food groups such as potatoes and pasta (Table I).

The reduction of food waste and correct meal portioning of specific food products are important because when they align and work together they can reduce food waste. This means data collected from consumers regarding what they consider to be the correct portion size in a meal is exceptionally valuable in waste reduction actions and it is rarely done. Obtaining such data is a challenge future research into food waste will need to address so that it can be transferred to food product development operations for maximum impact. The data collected here does not consider correct portion size data specifically but it does indicate its importance. The Austrian research reported here has shown that the fresh food thrown away per household per person for this sample was 37.48 kg each year while the frozen food thrown away per household per person was 6.46 kg and per year. The nutritional losses associated with food waste have yet to be fully characterised but they are an important component of food waste projections (Halloran *et al.*, 2014).

While we can determine the environmental impact of consuming foods in terms of their carbon footprint, it is the impact of wasting foods as an outcome of consumption that concerns us here. This is important because assessment of the environmental value of foods requires considerable investment of finance, knowledge and skills. It seems futile to make this investment if the assessed foods are wasted downstream in the food supply chain as they are prepared and consumed. New supply chain models are required to promote the value of reducing food waste and guide processes such as freezing that can reduce food waste. The data presented in Figure 1, clearly demonstrate a means to reduce the environmental impact of the food we choose to eat by reducing waste if frozen and freezing options are considered. The difficulty is that consumers choose foods based on what they like and this frequently changes, the choices made will rarely consider the impact of high level issues such as climate change but food waste reduction will be considered. This is because there is a very clear financial benefit to eliminating household food waste.

Current carbon footprinting methods show us that agri-production and global distribution can be the least of our problems because food wastage can be up to 20 per cent of food purchases and food losses across the supply chain can be far greater than this (Foster *et al.*, 2014). It is difficult to communicate such sustainability trade-offs in consumer arenas because debates are too complex to be made at the point of purchase. This is partly because carbon footprinting results are extremely variable due to the diversity of different food production systems and this has been tackled by developing certifications that target many sustainability goals. These have changed consumption of food by highlighting specific issues so that more ethical purchases are made such as those concerned with sustainable fishing, rainforest produce and so on. But it is day-to-day food waste at home and in supply chains that can make any diet unsustainable regardless of food certification used. Different preservation formats can reduce food waste and in the case of frozen food we know it can be reduced with respect to fresh foods because less of it is thrown away. There is no evidence that the nutritional values of frozen foods are any different to fresh foods if robust quality standards are in place from farm to fork. The nutritional losses resulting from food waste are significant and it is important to develop a food supply chain that is not losing these resources through wastage. There is not currently a certification that shows food produced with less waste or the use of food products that result in less waste and it is evident that there is a requirement to at least highlight the value of reducing consumer food waste. Food certification schemes that take household food waste reduction into account must be a future consideration in food and drink fast-moving consumer goods.

These ideas lead us to summarise the research presented here as a decision matrix model (Table II). The decision matrix highlights the major themes of consumer food waste reduction using frozen foods or freezing foods in households. It is proposed that such a matrix can be used to help food technologists guide the development of products with respect to preservation format and household food waste reduction. What is evident from the decision matrix analysis is a requirement to highlight the value of food preservation in reducing household food waste in the consumer space. This can be achieved by communicating through food companies’ Corporate Social Responsibility programmes as well as interventions that improve culinary knowledge in households. There are several emerging methods for achieving these interventions including digital applications that aim to reduce food waste and social media communications by creating consumer interest movements. It is important that food waste reduction initiatives integrate with these communication methods that consumers use (Martindale, 2017).

## Research conclusion

The research reported here shows purchased fresh foods have a six-fold greater food waste compared to purchased frozen food in a survey of 2,800 Austrian households. The research supports previous research conducted in the UK where a 47 per cent food waste reduction was demonstrated for frozen foods compared to fresh foods. This relationship shows maximal resource use is achieved for frozen food products that are manufactured for the convenience of being included in meals. The conclusion is that food manufacturers, food retailers and policy makers must consider the role of food preservation in delivering a sustainable diet. The decision matrix approach here provides initial guidance in new product development a basis for doing this and it is supported by data sets that have now been obtained in the Austrian and UK markets.

## Figures and Tables

**Table I:** The ratio of fresh to frozen food group waste for 2,800 Austrian households for the food product groups assessed

	Percentage of fresh food purchase wasted	Percentage of frozen food purchase wasted	Fresh to frozen food waste ratio
Fruit	6.2	0.6	10.3
Vegetables	5.5	1.4	3.9
Potatoes	3.9	0.5	7.8
Pasta	1.7	0.5	3.4
Meat	2.8	1.4	2.0
Fish	0.6	0.7	0.9

**Table II:** The decision matrix used to define the use of food preservation to reduce consumer food waste

Defining issues	Intervention issues identified by alternate and specific terms	Qualifier and outcome identifiers
Is frozen or freezing suitable for the food	Is the food material is suitable? Is the frozen market realistic (requiring market research)? Continuity of supply is required (e.g. to allow eating out-of-season)	LCA metrics can be used to improve the communication of environmental impact (e.g. the Carbon Footprint of a product)
How do you know it will reduce food waste	There is a fresh equivalent Current volumes of food waste need to be reduced Supply format provides convenience	There is currently a lack of tools to provide consumer advice. The research presented here helps to identify the benefits of preserving foods by freezing
How are consumption trends identified	Consumers must be familiar with product format. They may not typically use frozen formats	Peer review research studies must be used
How do we change behaviours when more sustainable ones are identified	Feedback from consumers will determine efficacy of using freezing as a preservation method	There is currently a lack of tools to provide consumer guidance A need for more robust methods to demonstrate specific food preparations can result in less waste
